# Direct assessment of tropical tuna abundance from their associative behaviour around floating objects

**DOI:** 10.1098/rspb.2024.1132

**Published:** 2024-08-21

**Authors:** Yannick Baidai, Amaël Dupaix, Laurent Dagorn, Daniel Gaertner, Jean-Louis Deneubourg, Antoine Duparc, Manuela Capello

**Affiliations:** ^1^ African Marine Expertises, Abidjan 04 BP 2991, Côte d’Ivoire; ^2^ MARBEC, Univ. Montpellier, CNRS, Ifremer, IRD, Sète, France; ^3^ Unit of Social Ecology, Université Libre de Bruxelles (ULB), Brussels, Belgium

**Keywords:** direct abundance index, associative behaviour, tropical tunas, echosounder buoys

## Abstract

Managing populations of wild harvested species requires the ability to regularly provide accurate abundance assessments. For most marine species, changes in abundance can only be monitored indirectly, using methods reliant on harvest-based indices, with significant inherent limitations surrounding the estimation and standardization of harvest effort. Tropical tunas are some of the most exploited marine species in the world and are among several species in critical need of alternative methods for estimating abundance. Addressing this concern, we developed the Associative Behaviour-Based abundance Index (ABBI), designed to provide direct abundance estimates for animals, which exhibit an associative behaviour with aggregation sites. Its implementation in the western Indian Ocean on skipjack tuna (*Katsuwonus pelamis*), yellowfin tuna (*Thunnus albacares*) and bigeye tuna (*Thunnus obesus*), revealed similar trajectories in their relative abundance. The ABBI stands as a potentially promising alternative to enhance traditional tropical tuna stock assessments methods, as well as a new opportunity to assess the abundance of other wild species that display an associative behaviour with physical structures found in their natural environment.

## 1. Introduction

Abundance is undeniably one of the most fundamental ecological parameters for decision making in the management and conservation of harvested species [1]. However, obtaining reliable abundance estimates for animal populations is typically problematic, especially for highly mobile species with extensive habitats [2]. In many cases, scientifically designed surveys have insufficient spatial and temporal coverage to achieve a comprehensive assessment of species abundance [3], largely due to budget or technical constraints [4]. When direct measurements of population size prove too challenging, indirect measures, which track changes in the overall population trends through proxies of their abundance, are commonly used to monitor wild species [5]. For commercially important fish species, catch and effort data are often the primary source of information used to monitor temporal changes in population size [6,7]. However, these data sources generally suffer from major limitations relating to the unknown changes in fish catchability and fishing efficiency, which both introduce substantial uncertainties into assessments of the species abundance trends [8]. For many fisheries, the development of alternative population assessment methods has become a critical challenge for ensuring their sustainable management [9].

With their extensive oceanic distribution and highly migratory behaviour, tropical tunas are a typical example of an exploited marine species group in critical need of alternative methods for estimating abundance. Skipjack (*Katsuwonus pelamis*), yellowfin (*Thunnus albacares*) and bigeye tuna (*Thunnus obesus*) support one of the world’s largest and most valuable fisheries [10,11] and are of key socio-economic importance for both developed and developing countries [12]. However, it is currently estimated that 10% of the total tuna catch comes from overfished stocks and 4% from stocks at intermediate levels of abundance [10].

Currently, the most widely used abundance estimates used in tropical tuna fisheries in all oceans rely on the catch-per-unit-effort (CPUE) indices derived from annually collected catch and effort data from fisheries [13]. The CPUE measures the amount of catch relative to the fishing effort expended. The term ‘effort’ encompasses various aspects of fishing activity, such as the duration of fishing, the number of hooks used or other measures of the labour invested in the fishing process. The assessment of abundance based on the CPUE is reliant on the conceptual proportionality it shares with abundance through catchability: a constant that describes how effectively a given unit of fishing effort captures the species. However, there is broad agreement that the interpretation of abundance indices derived from CPUE can provide misleading signals about actual trends in tuna species abundance. These are largely attributed to variations in tuna catchability resulting from numerous factors distorting catch rates, which then require complex standardization processes [6–8,14,15].

Standardized longline CPUEs provide the primary relative abundance indices for the stock assessment of yellowfin and bigeye tuna in both the Atlantic and Indian Oceans, as well as in the Pacific Ocean (both the for the Western and the Eastern stocks). However, contraction of effort in specific regions over time and changes in fishing strategies within this fishery pose additional challenges for accurately assessing abundance indices through the longline fisheries data [7,16]. Furthermore, tuna longline fisheries only account for a negligible proportion of the total skipjack tuna catch in all oceans. Consequently, despite skipjack tuna being the most important commercial species of tropical tuna fisheries, reliable indices for their stock assessments remain critically deficient. The main challenges in deriving reliable CPUE indices from tropical tuna purse-seine fisheries data consist in accounting for the frequent integration of new technologies on-board, the industrial purse seine vessels and for the continuous evolution of their fishing strategies, which make effort estimates difficult to conduct [17,18]. Since the 1990s, the development of this fishery has relied heavily on the use of artificial floating objects (FOBs) known as drifting fish aggregating devices (DFADs). DFADs are specifically designed to facilitate the capture of tropical tunas by capitalizing on their natural associative behaviour which results in the formation of multi-species aggregations around FOBs in the open ocean [18–21]. From early on in their development, DFADs were equipped with technology to facilitate their remote tracking, as locating them was key for fishers [22]. Currently, they also all include satellite-linked echosounder buoys that provide remote and near real-time information on the aggregations of tuna beneath them [23]. DFADs now contribute to more than half of the global tuna catch [18,19], with skipjack tuna (between 20 and 60 cm [18,24,25]) accounting for the majority of associated catch (57–82%), followed by mostly small or juvenile yellowfin (14–25%) and bigeye tuna (4–28%), across an interquartile size range of 47–73 cm and 49–69 cm fork length (FL), respectively [19,26,27]. The non-random nature of DFAD fishing has caused major disruptions in traditional purse seine CPUE indices. Developed prior to the widespread use of DFADs, these indices considered the number of search days as a unit of effort and did not incorporate the influence of remote reception of information on DFAD positions and associated biomasses [28].

Mark-recapture techniques generally provide complementary pieces of information used in assessing tuna abundance and their stock status. Mark-recapture involve the capture, tagging and release of a subset of individuals from a population, followed by port sampling to determine the proportion of tagged individuals among recaptures, providing the basis for estimates of the total population size, and other important information on the biology and migration of tuna species [29,30]. Tagging programmes are a common tool used by scientists and tuna regional fisheries management organizations (RFMOs) to improve their understanding of tropical tuna stocks. However, factors such as post-release mortality from tagging, tag shedding [31–33], poor tag reporting [34] and unknown dispersal dynamics of tagged tuna within the population [35,36] bring several levels of uncertainty that underpin their use for abundance estimates. Large-scale tuna tagging initiatives are also hampered by logistical challenges, high costs and significant resource investments. As a result, in most tuna RFMOs, conventional tagging data can only be collected on an episodic basis.

More recently, advances in molecular genetics have introduced an innovative approach based on a modified mark-recapture framework where ‘recaptures’ are kin rather than individuals. The approach, known as close-kin mark recapture (CKMR), uses the kinship relationship between randomly sampled tunas to estimate spawning stock biomass [37–39]. Despite its significant potential, CKMR remains experimental for tropical tunas, with early studies highlighting the challenges associated with sample size requirements for accurate population estimates [40–42].

In summary, robust indices for stock assessments of tropical tuna species are notably lacking across all oceans. Of particular concern is the Indian Ocean, the second largest tuna fishing region in the world [43]. Over the past decades, there has been a notable rise in the use of DFADs in this region, influenced by a number of factors including the impact of piracy on purse seine operations, the increased catch rates on DFADs compared to free-swimming schools (i.e. sets on tuna schools that are not associated with FOBs) and the introduction of total allowable catch for yellowfin tuna since 2016 (IOTC Resolution 16/01), and for bigeye tuna since 2023 (IOTC Resolution 23/04) [27,44–46]. Recent assessments indicate that both the bigeye and yellowfin tuna populations in this ocean are overfished and experiencing overfishing [47]. These sustainability concerns are further compounded by persistent challenges related to ongoing uncertainties in catch estimates, CPUE indices, conventional tagging data and reliance on assumptions about spatial structure and connectivity, all of which significantly undermine stock assessments [42,48]. Consequently, the pursuit of alternative methods for estimating tuna abundance has been identified as a critical objective within the work plan of the Indian Ocean Tuna Commission (IOTC) [49].

Within this context, this study presents a novel methodology that exploits the associative behaviour of tropical tunas, quantified using data from satellite-linked echosounder buoys attached to DFADs [50] and electronic tagging experiments, to provide direct estimates of their abundance: the Associative Behaviour-Based abundance Index (ABBI). Implemented in the western Indian Ocean, the ABBI yields abundance estimates for the three major tropical tuna species that associate with FOBs, skipjack and yellowfin and bigeye tuna. In a broader context, the ABBI introduces a promising methodological framework for exploring population trends of various wild species that naturally exhibit associative behaviour with living and non-living aggregation sites, based on a limited set of metrics describing their associative dynamics.

## 2. Methods

### (a) The ABBI framework

The associative behaviour of tropical tuna species implies that, at any given time *t*, the overall abundance (*N*(*t*)) in an area with *p* FOBs results from the sum of the abundances of their associated component ( $X_{a}\left(t\right)$ ) located nearby FOBs and unassociated ( $X_{u}\left(t\right)$ ) component, located away from FOBs (i.e. free-swimming tuna schools).


(2.1) $$\begin{array}{c} N\left(t\right)=X_{a}\left(t\right)+\;X_{u}\left(t\right)\; \end{array}\;\;$$

Within a given study region and time period, the estimated size of the associated component of the tuna population ( $\hat{X_{a}}$ ) can be estimated as follows:


(2.2) $$\begin{array}{c} \hat{X_{a}}\;=\;\hat{m}\hat{f}\hat{p} \end{array}$$

Where $\hat{m}$ is the estimated average tuna biomass associated with a FOB, $\hat{f}$ represents the estimated proportion of FOBs with tuna aggregations and $\hat{p}$ the estimated number of FOBs in the region/period of interest. Previous studies [51] demonstrated that the ratio between the estimated size of the associated component and the estimated total population ( $\hat{N}$ ) can be derived by measuring the uninterrupted period of time that tunas spend either associated with, or unassociated from a FOB, i.e. the average continuous residence time (CRT) and the average continuous absence time (CAT):


(2.3) $$\begin{array}{c} \frac{\hat{X_{a}}}{\hat{N}}=\frac{CRT\;}{CRT+\;CAT}\; \end{array}$$

Combining equations (2.1)–(2.3), the total population can then be estimated as follows:


(2.4) $$\begin{array}{c} \hat{N}=\;\hat{m}\hat{f}\hat{p}\left(1+\frac{CAT}{CRT}\right) \end{array}$$

As the associative dynamics of tuna [52,53] are dependent on species and size, equation (2.4) is applicable on a species and size classes basis, where the same associative behaviour with FOBs is expected. Furthermore, considering the above equations, the size of the unassociated component of the tuna population can be estimated as follows:


(2.5) $$\begin{array}{c} {\hat{X}}_{u}=\frac{CAT}{CRT}\hat{m}\hat{f}\hat{p}\; \end{array}$$

**Figure 1.**
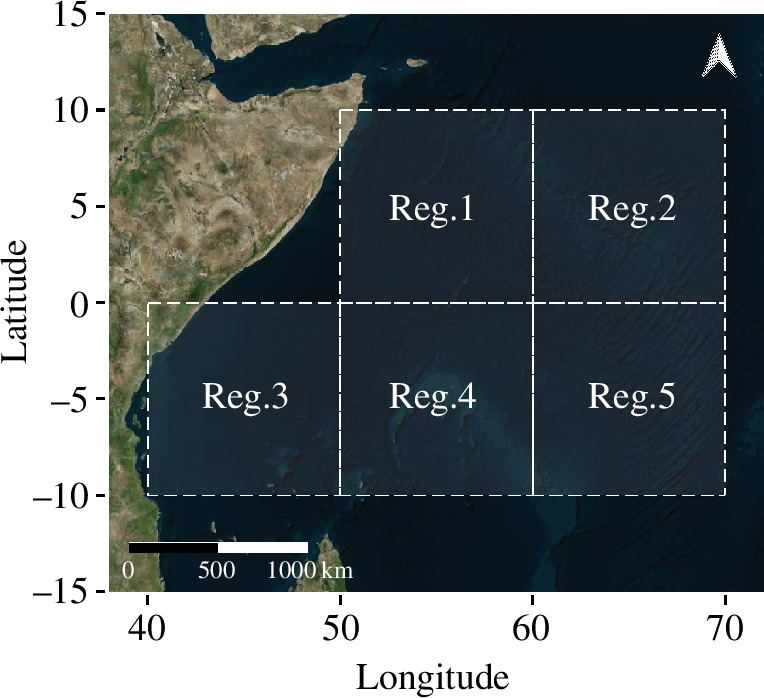
Spatial stratification of the study area. The abundance estimates were conducted individually in each of the five regions identified by the abbreviation ‘Reg.’. Then, an average abundance index for the entire study area was derived.

Estimating abundance from the ABBI framework as provided by equation (2.4) requires a set of input data which is currently available for the three major tropical tuna in the Indian ocean. The abundance estimates were conducted in the western Indian Ocean, between 2013 and 2021, using a spatio-temporal stratification of 10° × 10° and quarter-year (figure 1). ABBI indices of abundance were estimated within each stratum for each species and population components (estimated total population (equation 2.4); estimated size of the associated component (equation 2.2); estimated size of the unassociated population (equation 2.5)). For yellowfin and bigeye tuna, only the component of the population related to small individuals (weight under 10 kg) was considered. This corresponds to the main size category caught at FOBs (see electronic supplementary material, figure S1) and for which data on residence and absence times have been measured [54].

For each species, ABBI abundance indices over the entire study area were estimated from the average of the quarterly ABBI indices obtained over the available strata, resulting in a final index representing the average abundance per 10° × 10° square, quarter and species. Finally, ABBI relative abundance indices were provided using the ABBI abundance indices of the first available quarter of 2013 (i.e. the first quarter in which the input dataset for the implementation of ABBI was available) as reference.

### (b) Estimated number of floating objects ( $\hat{p}$ )

Drifting FOBs considered in this study consist of two main categories: DFADs and ‘other objects’. The DFAD category contains man-made FOBs specifically designed and deployed by fishers to encourage tuna aggregations, while the ‘other objects’ category includes natural drifting objects (*e.g*. branches or logs) and anthropogenic debris (artificial objects resulting from human activities related or not to fishing). Due to the availability of DFAD location data changing over time, the estimation of the total number of FOBs within each time–area unit followed two different approaches.

From 2013 to 2019, time series of the total number of FOBs were constructed from the echosounder buoys database hosted by the Observatoire des Ecosystèmes Pélagiques Tropicaux Exploités (Ob7/IRD/MARBEC). The dataset consists of position data collected by the ‘Marine Instruments’ echosounder buoys deployed on DFADs by the French fleet in the Indian Ocean. First, the average number of buoys was calculated from this database, for each spatio-temporal stratum (10° er quarter). Then, total numbers of FOBs (DFADs and ‘other objects’) in the different strata were estimated by applying multiplication factors taking into account the number of DFADs belonging to other purse seine fleets, as well as the ‘other objects’ category (see details in electronic supplementary material, S1).

From 2020 to 2021, the estimation of FOBs number have benefitted from the recent availability of echosounder buoys data from tuna purse-seine vessels provided by the IOTC Secretariat [55]. This dataset consists of the monthly average of the number of operational buoys (i.e. echosounder buoys that remotely transmit their position) for each 1° × 1° cell of the Indian Ocean. The total number of FOBs in the different strata was then estimated by aggregating the data according to the level considered in the spatio-temporal strata of the study, and applying a raising factor to account for the number of ‘other objects’. Electronic supplementary material, S1 and figure S2 provide complete details of the methodology and estimates of the number of FOBs in the study area, respectively.

### (c) Estimated FOB-associated tuna biomass ( $\hat{m}$ )

The average biomasses of skipjack, small yellowfin and small bigeye tunas (size category under 10 kg) around a FOB were derived from purse seine catch-per-set data reported in the vessel logbooks of the French fleet (electronic supplementary material, table S1). In order to improve the accuracy of the estimated catches, FOB-associated catches-per-set reported in vessel logbooks were corrected using a dedicated procedure referred to as levels 1 and 2 of the T3 processing [56–58]. Level 1 adjusts the catch-per-set values declared in vessel logbooks using landing notes, to improve the accuracy of catch estimates provided by skippers. Level 2 estimates the species and size compositions of FOB sets based on port sampling data.

Since landing notes were available for all fishing trips, Level 1 was applied to correct the reported catch-per-set of all FOB sets recorded in vessel logbook data. Level 2, on the other hand, was applied only to the FOB sets conducted during the fishing trips that were sampled at landing. These FOB sets are referred to as ‘sampled FOB sets’.

Species compositions (i.e. percentages of catches by species and size category in the sampled FOB sets) were averaged by stratum, with a minimum threshold of 20 available sampled FOB sets per strata. Where species composition values were missing for a given stratum, they were generated using their corresponding estimated marginal means (aka least-squares means), following a procedure described in the electronic supplementary material, S2.

Finally, the average biomasses of skipjack, small yellowfin and small bigeye tunas (size category under 10 kg) associated with a FOB ( $\hat{m}$ ) were calculated for each stratum by multiplying the average catch-per-set of all FOBs (including both sampled and not-sampled sets, all adjusted through the Level 1 of the T3 processing) by the average species composition. Only the strata with at least 20 FOB sets (including both sampled and not-sampled sets) were retained for the estimation of $\hat{m}$ and the derivation of the ABBI indices.

The catch and species composition data provided by the Ob7 were collected under the Data Collection Framework (Reg. 2017/1004 and 2016/1251) funded by IRD and the European Union. Electronic supplementary material, figure S3 shows the time series of the FOB-associated biomasses ( $\hat{m})$ obtained for each of the three species and size categories, across the various spatial strata considered.

### (d) Estimated proportion of FOBs occupied by tunas ( $\hat{f}$ )

The proportions of FOBs occupied by tuna were estimated from the acoustic data collected by the Marine Instruments M3I satellite-linked echosounder buoys deployed on FOBs monitored by the French tuna purse seine fleet in the western Indian Ocean (also available from the OB7 hosted echosounder buoys database). This model of buoy has an integrated echosounder device (frequency 50 kHz, power 500 W, beam angle of 36°), which provides acoustic information on the biomass associated with the FOB, with a detection threshold of 1 tonne [50]. This detection threshold was considered satisfactory as it ensured consistency between the datasets of estimated FOB occupancy rate ( $\hat{f}$ ) and associated biomass ( $\hat{m}$ ) estimated from catch data because fishing operations are seldom carried out on FOBs with less than one tonne of associated tuna biomass (approximately 0.5% of the fishing sets performed by French tuna purse seine fleet in the Indian Ocean). Processing acoustic data from buoys using a machine learning algorithm, whose accuracy has been demonstrated (85%) in the Indian Ocean [50], allowed for geolocated times series of tuna presence/absence data under FOBs. Then, the initial segments immediately following the deployment of the FOBs, consisting of tuna absence data, were excluded from the analysis as they reflect the FAD colonization period [59].

Daily presence/absence data were used to derive the estimated proportion of FOBs with tuna associated ( $\hat{f}$ ). This was expressed as the number of FOBs (equipped by an M3I buoy) occupied by tunas, divided by the total number of M3I buoys available in the database, calculated on a daily basis. A threshold of at least 30 available M3I buoys per day was considered when calculating the daily proportion of FOBs occupied by tuna across the spatial strata. Electronic supplementary material, table S1 provides the average daily numbers of available M3I buoys over the full study area for each quarter.

The buoy technology remains limited in its ability to discriminate between the various tuna species [23]. Nevertheless, this limitation can be overcome to achieve finer-grained details on FOB occupancy at a species level. This is achieved by combining the estimated fraction of FOBs occupied by tunas ( $\hat{f}$ ) with the rate of occurrence of each species in FOB aggregations obtained from FOB catches and sampling data, according to the following equation:


(2.6) $$\begin{array}{c} \hat{f_{i}}=\hat{f}.\hat{\eta_{i}} \end{array}$$

where $\hat{f_{i}}$ denotes for the estimated proportion of FOBs occupied by the tuna species i , and $\hat{\eta_{i}}$ represents the ratio of the number of FOB associated sets that resulted in a catch greater than or equal to 1 tonne of the species in question, to the total number of FOB sets. This ratio was estimated on a quarterly basis, within each 10° × 10° spatial stratum, using data from port sampling programs. A minimum number of 20 available sampled fishing sets per strata was considered for the ratio calculation. Missing occurrence values for a given stratum were estimated from a binomial model using year, quarter and spatial strata as predictors (electronic supplementary material 3). The time series of $\hat{f}$ are presented in the electronic supplementary material, figure S4 for each of the three species.

### (e) Continuous residence time

The CRT values were provided by the acoustic tagging experiments carried out around DFADs in the study area by Govinden *et al*. [54], whose estimates of the CRTs of skipjack (49 cm FL on average) and small yellowfin and bigeye tunas (62 and 53 cm FL on average, respectively) around a DFAD were 4.6 ± 4.8 days, 6.7 ± 7.8 days, and 7.6 ± 7.2 days, respectively.

### (f) Continuous absence time

At the time of the study, only CRTs were measured for the three species on DFADs. However, acoustic tagging experiments conducted in arrays of anchored Fish Aggregating Devices (AFADs) showed that CATs decrease for decreasing distances among AFADs, due to an increased AFAD encounter rate by tuna at higher AFAD densities [60]. Based on these findings, the following Ansatz relating the average CAT to the number of FOBs ( $\hat{p}$ ) was used:


(2.7) $$\begin{array}{c} CAT=\frac{1}{\phi \hat{p}} \end{array}$$

where *ϕ* is a parameter that depends on the probability of associating to one of the estimated $\hat{p}$ FOBs. To assess the sensitivity of the ABBI to *ϕ* values, the range of 2 × 10^−5^ and 6 × 10^−5^ that produces average CATs ranging between 10 and 30 days, consistent with the findings from acoustic tagging studies [60–62], was considered for the abundance assessments (electronic supplementary material, figure S5). A detailed section on the significance and the order of magnitude of *ϕ* is provided in electronic supplementary material 5.

## 3. Results

**Figure 2.**
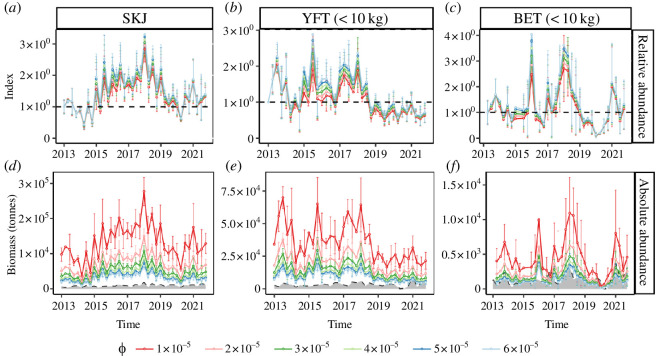
Estimates of the ABBI for the total populations (associated and non-associated components) of the three major tropical tuna species in the western Indian Ocean under different values of *ϕ* (±s.e.). (*a–c*) Time series of the average relative abundances estimated over the five 10° × 10° regions shown in figure 1. (*d–f*) Time series of the average absolute abundances estimated over the five 10° × 10° regions shown in figure 1. The shaded areas correspond to the average catches per 10° × 10° square of all the fishing gears targeting the species and life stages of the tunas considered, in the western Indian Ocean. SKJ: skipjack tuna, YFT (<10 kg): yellowfin tuna under 10 kg and BET (<10 kg): bigeye tuna under 10 kg.

In the Western Indian Ocean, the ABBI estimates revealed similar trajectories in the abundances of the three tuna species (skipjack, yellowfin and bigeye tuna) considered in this study, both in terms of total abundance (figure 2; electronic supplementary material, figure S6) and associated versus non-associated components (electronic supplementary material, figure S7). Overall, the abundance of the three species experienced two minima, both followed by a steady increase. The first minimum occurred in 2014, and was particularly marked for skipjack and yellowfin tuna. The second minimum, observed in 2020, predominantly affected bigeye (figure 2).

**Figure 3.**
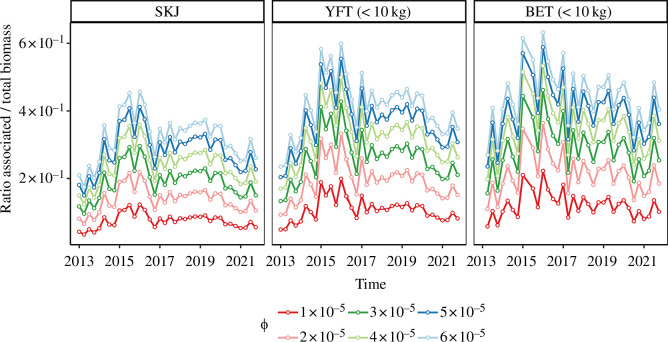
Time series of the ratio of the average biomass of the associated component to the total average biomass estimated for different values of ϕ corresponding to different ranges of CAT. All averages were estimated over the five 10° × 10° regions shown in figure 1. SKJ: skipjack tuna, YFT (<10 kg): yellowfin tuna under 10 kg and BET (<10 kg): bigeye tuna under 10 kg.

When examined in relative terms (figure 2*a–c*), the ABBI showed very low sensitivity to the values of the parameter (*ϕ*) used in setting the ranges of CAT for all species. In contrast, the orders of magnitude of the absolute ABBI estimates (figure 2*d–f*) remained closely linked to the ranges of CAT used. For average CATs, expected to be between 10 and 30 days (corresponding to *ϕ* values of 6 × 10^−5^ and 2 × 10^−5^, respectively), the ABBI estimates indicated average skipjack biomasses ranging between 30 700 ± 12 500 tonnes and 71 500 ± 27 300 tonnes per 10° × 10° square, over the study area and period. For the same area and period, the ABBI estimates for the average biomasses of small yellowfin tuna ranged from 9300 ± 4100 tonnes to 20 000 ± 8300 tonnes per 10° × 10° square and from 1200 ± 800 tonnes to 2500 ± 1500 tonnes per 10° × 10° square for small bigeye tuna. Globally, the biomass levels of small bigeye tuna appeared to be around eight times lower than those of yellowfin tuna of the same size class. Furthermore, the ratios between the associated and the total population revealed general increasing trends for all three species, with maximum values reached between 2015 and 2016 (figure 3).

## 4. Discussion

We provide an innovative framework for estimating the abundance of tropical tuna populations based on their associative behaviour with FOBs and the large-scale acoustic datasets collected through satellite-linked echosounder buoys deployed by fishers. The ABBI approach allows for both an abundance assessment of the populations of interest and the calculation of its associated and non-associated components. It presents a promising opportunity that circumvents the specific challenges associated with estimating fishing effort, which hinder CPUE-based methods for assessing tuna abundance. While based on a modelling framework that utilizes the associative behaviour displayed by animals at aggregation sites, the ABBI does not require an understanding of the causative factors underlying this behaviour [51]. Conceptually, the behaviour of the animal includes the various biotic and abiotic factors that modulate its residence and absence times in the array of aggregation sites, and on which relies the calculation of the ratio between the ‘observable’ component of the population (i.e. the one that can be easily sampled, namely the associated population) and the total population.

For tropical tunas, the data inputs for the ABBI framework have been facilitated through several technological developments in recent decades. These include innovations in electronic tagging and tracking for studying fish ecology [54,63], and in the tuna purse seine fishery, with the development of echosounder buoys. The utilization of the large-scale location and acoustic datasets collected by echosounder buoys has created the unprecedented ability to monitor tuna aggregation dynamics at FOBs [59], and thus constituted a critical element for the effective implementation of the ABBI methodology for these species. Two of the five key metrics used in the ABBI approach, namely the total number ( $\hat{p}$ ) and the proportion of FOBs inhabited by tuna ( $\hat{f}$ ), are derived from these devices. The current buoy models are also designed to provide estimates of the associated biomass underneath the DFADs ( $\hat{m}$ ). However, at present, these estimates have been shown to be insufficiently accurate to be used in scientific studies [50]. To circumvent these limits, in this study, the associated biomass was estimated from purse-seine catch data under DFADs. The use of catch data to estimate tuna aggregation sizes can also imply potential biases, due to the increasing fishing efficiency of purse seiners resulting from the use of echosounder buoys. Namely, the remote monitoring of the DFAD-associated biomass could result in the selection of larger tuna aggregations, which could in turn bias the ABBI abundance estimates. However, recent studies indicate that DFAD catches typically increase by only 2–2.5 tonnes per set (equivalent to approximately 10% of the average catch-per-set at DFADs) when fishing operations are conducted on the vessel’s owned DFADs equipped with an echosounder buoy [25]. Furthermore, the fishing strategies employed by the French purse seine fleet, whose data were used in the study, still involve a high proportion of sets on foreign DFADs (i.e. DFADs that do not belong to the vessel and are therefore not monitored through echosounder buoys) during the study period, thereby mitigating this bias to a minor extent within the case study. Given the rapid and continuous advances in buoy technology [22], it is reasonable to expect that current buoy models will evolve towards models with enhanced detection and estimation performance, enabling direct estimation of DFAD-associated biomasses and removing the limitations from catch data.

Here, the ABBI framework illustrates the important contribution that unconventional data sources and technologies such as echosounder buoys and electronic tagging can make towards improving the inputs in fish stock assessments. Tuna CRTs are straightforward to measure using existing acoustic tagging technologies, where DFADs are instrumented with an acoustic receiver (hydrophone) to record the presence of acoustically tagged fish (i.e. fish equipped with an acoustic emitter) in their proximity. In this study, we utilized average CRT values recorded for each species during acoustic tagging experiments on DFADs located in the study region [53]. Past acoustic tagging experiments conducted in different areas and FAD arrays have shown that CRTs can vary not only according to the tuna species and size category [52,53], but also with the FOB density [60]. However, the magnitude of this variation remains relatively small compared with those observed for the CATs, for the tuna species and life stages considered in this paper [54,63–66]. Additionally, this study does not consider other possible seasonal and/or regional variations of CRTs, that may originate from local environmental variables (including tuna abundance), since the available acoustic tagging data collected on DFADs [54,63] do not present a sufficient spatial and temporal coverage allow to characterize them. The lack of comprehensive CRT data limits the ability of the ABBI to fully capture the complexity of the behaviour of the animal population being studied. For tropical tunas, the substantial differences in magnitude between documented CRTs and plausible ranges of CATs suggest a minimal influence of CRT variability on ABBI performance (see the sensitivity analysis to different values of CRT in the electronic supplementary material 5 and figure S8). However, this limitation underscores the need for extensive and systematic electronic tagging programs. Regular and large-scale electronic tagging programmes will be essential to quantify such variations and to incorporate possible seasonal and regional changes of tuna associative behaviour within the ABBI approach.

Furthermore, quantifying CATs remains challenging in DFAD arrays but is relatively easier in arrays of AFADs [61,67]. Results from electronic tagging experiments at anchored FADs coupled to models indicate decreasing CATs recorded for increasing FOB densities [60,61]. Consequently, in the absence of CATs recorded in arrays of drifting DFADs, an inverse proportionality relation was considered in the derivation of the ABBI index (equation 2.7). The robustness of the approach was tested considering different values of the ϕ parameter (equation 2.7), taking into account plausible ranges of CATs recorded in previous acoustic tagging studies. Globally, the relative ABBI indices show little sensitivity to variations of the ϕ parameters (figure 2*a–c*). On the other hand, the ABBI absolute abundance indices show higher sensitivities to *ϕ*. Although the ABBI is designed to produce absolute abundance estimates, the robustness of these estimates for tropical tuna is significantly weakened by the assumptions surrounding the CAT values, currently representing one of the major constraints within the approach. Increased efforts for additional field tagging studies (providing CAT values in arrays of DFADs) or further research investigating the explicit relationship between this metric and FOB densities would be valuable in providing more accurate estimates of tuna absolute abundance. However, it is noteworthy that, with the exception of bigeye tuna, the ABBI estimates of absolute abundance provided in this study become increasingly consistent as *ϕ* values decrease. This is illustrated by the fact that they significantly exceed the total catches reported in the same study area and time period for all gears, which currently serve as the only available lower bound for tuna abundance. This would imply higher tuna absence times with drifting FADs than with anchored FADs, the range of which was considered in this study in the absence of better scientific available data.

Accurate information on the spatial distribution and densities of FOBs is also critical. As DFADs are the predominant class of FOBs [68], the geolocation data from their echosounder buoys [22,23,69] could allow the reconstruction of the densities of FOBs at fine spatial and temporal scales. Since 2020, data on the number of operational buoys (i.e. echosounder buoys transmitting their position and acoustic data to the purse seiners) have been collected by the Secretariat of the IOTC and made available at a scale of 1° per month, ensuring more reliable estimates of the density of FOBs (IOTC Resolution 19/01). However, their availability was relatively limited for the time period considered in this study and DFAD densities were estimated from other data sources prior to 2020 (see electronic supplementary material, S1). Remarkably, despite the use of two different methods, the global time series of the number of FOBs exhibits consistent trends across the full study period (electronic supplementary material, figure S2), with no major breaks occurring around 2020. However, a higher seasonal variability can be observed for the years prior to 2020. These seasonal patterns observed in the French fleet [70], which constitute the exclusive data source for estimating the total number of FOBs prior to 2020, may have introduced additional variability in the abundance estimates. This aspect deserves attention, particularly considering that tropical tuna stock assessments are typically conducted on a quarterly time-step. However, this artificial variability can easily be smoothed by supplementing the dataset with additional buoys from other fleets in the IOTC database, as demonstrated for the period after 2020.

Another important aspect in the derivation of the ABBI index is the choice of the spatio-temporal strata. The 10° per quarter scale chosen in this study represents a trade-off between having enough data per stratum to estimate each of the relevant variables (sampled species composition, number of FOBs and number of M3I echosounder buoys to estimate m , p and f in equation 2.6) and ensuring homogeneous conditions (including the number of FOBs) at the spatio-temporal scale considered. Despite in real systems homogeneity is difficult to achieve, the violation of this condition can be tested by inspecting relevant behavioural metrics, such as the fraction of occupied FOBs. If major heterogeneities occurred within the strata and affected tuna behaviour, we would expect to observe non-stationary trends in this metric. Stationarity tests on the time series of the proportion of inhabited FOBs demonstrated that, across the spatial strata considered, this proportion is stationary for each stratum (see electronic supplementary material 6 and figure S8), supporting the choice of the 10° per quarter scale for deriving ABBI.

Overall, while absolute abundance estimates derived from the ABBI are possible, their current accuracy is globally constrained by availability and uncertainties surrounding CAT data. Conversely, the relative index, which exhibits little variability to *ϕ*, appears to demonstrate greater robustness, especially concerning skipjack and yellowfin tuna. Comparison of this index with outputs from the Stock Synthesis 3 model (SS3) indicates significant correlations for these two species, as detailed in electronic supplementary material, S7 and figure S10.

Despite the relatively short length of the available time series, our findings indicate a significant temporal similarity between the abundance trends of skipjack, small yellowfin and small bigeye tunas in the Western Indian Ocean. These trends were characterized by two episodes of declining abundance in the populations of all three species. The first coincided with the general increase in the density of FOBs observed before 2015. The second has been ongoing since 2017–2018, despite a general stabilisation in the number of FOBs following successive limitation measures introduced by the IOTC (IOTC Resolution 15/08 superseded by 17/08, 18/08, 19/02). Besides the likely influence of environmental factors, the fishing mortality resulting from the DFAD purse seine fishery might well be among the key drivers of the observed synchronous population dynamics. As the estimated biomass levels for each of these species appear to be very different, but show similar temporal trends, such a result raises questions about the appropriateness of a species-specific management approach. For instance, in order to rebuild the Indian Ocean yellowfin tuna stock, which is overfished and subject to overfishing, the IOTC has implemented management measures since 2016, including catch limits for this species (Resolution 16/01 superseded by 17/01, 18/01, 19/01 and 21/01). For the tuna purse-seine fisheries, these measures forced a change in the fishing patterns, with a notable shift towards targeting skipjack tuna which are predominantly caught under FOBs [19]. A decrease in skipjack abundance resulting from this increased fishing pressure would thus imply an effect of similar magnitude on the small individuals of the other two species, since they all share similar abundance dynamics and are caught together when associated with FOBs. While skipjack are considered to be more resilient to fishing pressure [71], such a strategic shift could have greater disruptive impacts on the juveniles of the two other species due to the significant concerns that already surround the resilience of their stocks.

In summary, ABBI holds significant potential for improving abundance assessment of tropical tuna and, consequently stock assessments and fisheries management. It also represents a convenient methodological framework for studying the populations of all marine species that associate with FOBs [72]. These include pelagic sharks such as the vulnerable-listed silky shark, *Carcharhinus falciformis* [73], and the critically endangered oceanic whitetip shark, *C. longimanus* [74], as well as other bony fish species [75] which currently receive little or no attention from tuna RMFOs. Finally, the potential of ABBI is not limited to marine species. It can be extended to a broader scope, covering various species which exhibit associative behaviour around non-living or living entities (e.g. seabirds and cetaceans [76], facultative parasites and their hosts [77], fish and drifting algae [78], jellyfish [79] or whale sharks [80], etc.), and thus unlocks new prospects for population assessments of a wide range of wild animal species.

## Data Availability

The data used for the ABBI model were collected through the Data Collection Framework (Reg 2017/1004 and 2016/1251), funded by the IRD, the European Union, and France. They can be accessed upon request via the IRD Observatory of Tropical Ecosystems (Ob7) through the dedicated website: https://ob7-ird.science/les-donnees/. Supplementary material is available online [81].

## References

[1] Pereira HM *et al* . 2013 Essential biodiversity variables. Science **339** , 277–278. (10.1126/science.1229931) 23329036

[2] Boyd C , Barlow J , Becker EA , Forney KA , Gerrodette T , Moore JE , Punt AE . 2018 Estimation of population size and trends for highly mobile species with dynamic spatial distributions. Divers. Distrib. **24** , 1–12. (10.1111/ddi.12663)

[3] Hilborn R , Walters CJ . 1992 Quantitative fisheries stock assessment: choice, dynamics and uncertainty. Boston, MA: Springer Science & Business Media. (10.1007/978-1-4615-3598-0)

[4] Dennis D , Plagányi É , Van Putten I , Hutton T , Pascoe S . 2015 Cost benefit of fishery-independent surveys: are they worth the money? Mar. Policy **58** , 108–115. (10.1016/j.marpol.2015.04.016)

[5] Gibbs JP , Droege S , Eagle P . 1998 Monitoring populations of plants and animals. Bioscience **48** , 935–940. (10.2307/1313297)

[6] Harley SJ , Myers RA , Dunn A . 2001 Is catch-per-unit-effort proportional to abundance? Can. J. Fish. Aquat. Sci. **58** , 1760–1772. (10.1139/f01-112)

[7] Maunder MN , Sibert JR , Fonteneau A , Hampton J , Kleiber P , Harley SJ . 2006 Interpreting catch per unit effort data to assess the status of individual stocks and communities. ICES J. Mar. Sci. **63** , 1373–1385. (10.1016/j.icesjms.2006.05.008)

[8] Maunder MN , Punt AE . 2004 Standardizing catch and effort data: a review of recent approaches. Fish. Res. **70** , 141–159. (10.1016/j.fishres.2004.08.002)

[9] Maunder MN , Piner KR . 2015 Contemporary fisheries stock assessment: many issues still remain. ICES J. Mar. Sci. **72** , 7–18. (10.1093/icesjms/fsu015)

[10] ISSF . 2024 Status of the world fisheries for tuna. ISSF Tech. Rep. 2024-02. International Seafood Sustainability Foundation. See https://www.iss-foundation.org/about-issf/what-we-publish/issf-documents/issf-2024-02-status-of-the-world-fisheries-for-tuna-march-2024/.

[11] FAO . The state of world fisheries and aquaculture 2022. Food and Agriculture Organization of the United Nations. See http://www.fao.org/documents/card/en/c/cc0461en.

[12] Bell JD , Kronen M , Vunisea A , Nash WJ , Keeble G , Demmke A , Pontifex S , Andréfouët S . 2009 Planning the use of fish for food security in the pacific. Mar. Policy **33** , 64–76. (10.1016/j.marpol.2008.04.002)

[13] Fu D . 2023 Indian Ocean skipjack tuna stock assessment 1950-2022 (Stock Synthesis). IOTC–2023–WPTT25–09. Indian Ocean Tuna Commission. See http://www.iotc.org/files/proceedings/2012/wptt/IOTC-2012-WPTT14-29 Rev_1.pdf.

[14] Bishop J . 2006 Standardizing fishery-dependent catch and effort data in complex fisheries with technology change. Rev. Fish Biol. Fish. **16** , 21–38. (10.1007/s11160-006-0004-9)

[15] Ye Y , Dennis D . 2009 How reliable are the abundance indices derived from commercial catch–effort standardization? Can. J. Fish. Aquat. Sci. **66** , 1169–1178. (10.1139/F09-070)

[16] Utama FW , Hoenner X , Hardesty BD , Peel D , Ford JH , Adams V , Wilcox C . 2021 Estimating fishing effort and spatio-temporal distribution of longline vessels in the indian ocean. Front. Mar. Sci. **8** , 8:671036. (10.3389/fmars.2021.671036)

[17] Torres-Irineo E , Gaertner D , Chassot E , Dreyfus-León M. 2014 Changes in fishing power and fishing strategies driven by new technologies: the case of tropical tuna purse seiners in the eastern Atlantic Ocean. Fish. Res. 155, 10-19. (10.1016/j.fishres.2014.02.017)

[18] Fonteneau A , Chassot E , Bodin N. 2013 Global spatio-temporal patterns in tropical tuna purse seine fisheries on drifting fish aggregating devices (DFADs): taking a historical perspective to inform current challenges. Aquat. Living Resour. 26, 37-48. (10.1051/alr/2013046)

[19] Dagorn L , Holland KN , Restrepo V , Moreno G . 2013 Is it good or bad to fish with fads? what are the real impacts of the use of drifting fads on pelagic marine ecosystems? Fish Fish **14** , 391–415. (10.1111/j.1467-2979.2012.00478.x)

[20] Castro JJ , Santiago JA , Santana-Ortega AT . 2001 A general theory on fish aggregation to floating objects: an alternative to the meeting point hypothesis. Rev. Fish Biol. Fish **11** , 255–277. (10.1023/A:1020302414472)

[21] Fonteneau A , Ariz J , Gaertner D , Nordstrom V , Pallares P . 2000 Observed changes in the species composition of tuna schools in the gulf of guinea between 1981 and 1999, in relation with the fish aggregating device fishery. Aquatic Living Res. **13** , 253–257. (10.1016/S0990-7440(00)01054-8)

[22] Lopez J , Moreno G , Sancristobal I , Murua J . 2014 Evolution and current state of the technology of echo-sounder buoys used by spanish tropical tuna purse seiners in the Atlantic, Indian and Pacific oceans. Fish. Res. **155** , 127–137. (10.1016/j.fishres.2014.02.033)

[23] Moreno G , Boyra G , Sancristobal I , Itano D , Restrepo V . 2019 Towards acoustic discrimination of tropical tuna associated with fish aggregating devices. PLoS One **14** , e0216353. (10.1371/journal.pone.0216353) 31166986 PMC6550443

[24] Hidayat R , Zainuddin M . 2021 Characteristics of skipjack tuna fisheries using fad and non-fad methods: an important step for fisheries management in the gulf of bone and flores sea, indonesia. AACL. Bioflux **14** , 821–831. http://www.bioflux.com.ro/docs/2021.821-831.pdf

[25] Hidayat R , Zainuddin M , Mallawa A , Mustapha MA , Putri ARS . 2019 Comparing skipjack tuna catch and oceanographic conditions at FAD locations in the gulf of bone and makassar strait. IOP Conf. Ser. Earth Environ. Sci. **370** , 012038. (10.1088/1755-1315/370/1/012038)

[26] Phillips JS , Pilling GM , Leroy B , Evans K , Usu T , Lam CH , Schaefer KM , Nicol S . 2017 Revisiting the vulnerability of juvenile bigeye (Thunnus obesus) and yellowfin (T. albacares) tuna caught by purse-seine fisheries while associating with surface waters and floating objects. PLoS One **12** , 1–18. (10.1371/journal.pone.0179045) PMC5490998 28662091

[27] Davies TK , Mees CC , Milner-Gulland EJ . 2014 The past, present and future use of drifting fish aggregating devices (fads) in the indian ocean. Mar. Policy **45** , 163–170. (10.1016/j.marpol.2013.12.014)

[28] Wain G , Guéry L , Kaplan DM , Gaertner D . 2021 Quantifying the increase in fishing efficiency due to the use of drifting fads equipped with echosounders in tropical tuna purse seine fisheries. ICES J. Mar. Sci. **78** , 235–245. (10.1093/icesjms/fsaa216)

[29] Fonteneau A , Hallier JP . 2015 Fifty years of dart tag recoveries for tropical tuna: a global comparison of results for the western pacific, eastern pacific, atlantic, and indian oceans. Fish. Res. **163** , 7–22. (10.1016/j.fishres.2014.03.022)

[30] Eveson JP , Million J , Sardenne F , Le Croizier G . 2015 Estimating growth of tropical tunas in the indian ocean using tag-recapture data and otolith-based age estimates. Fish. Res. **163** , 58–68. (10.1016/j.fishres.2014.05.016)

[31] Hoyle SD , Leroy BM , Nicol SJ , Hampton WJ . 2015 Covariates of release mortality and tag loss in large-scale tuna tagging experiments. Fish. Res. **168** , 47–48. (10.1016/j.fishres.2015.03.006)

[32] Gaertner D , Goñi N , Amande J , Alayon PP , Gom FN , Pereira J , Addi E , Beare D . 2019 First estimate of tag-thedding for bigeye tuna in the atlantic ocean from ICCAT AOTTP data. Collect. Vol. Sci. Pap. ICCAT, pp. 1875–1880. See https://www.iccat.int/Documents/CVSP/CV075_2018/n_7/CV075071875.pdf.

[33] Katara I , Gaertner D , Billet N , Lopez J , Fonteneau A , Murua H , Baez JC . 2017 Standardisation of skipjack tuna CPUE for the EU purse seine fleet operating in the Indian Ocean. Work. In 19th Work. Party Trop. Tunas.

[34] Fromentin J . 2010 Tagging bluefin tuna in the mediterranean sea: challenge or mission: impossible?. Collect. Vol. Sci. Pap. ICCAT, pp. 812–821. See https://www.iccat.int/Documents/CVSP/CV065_2010/n_3/CV065030812.pdf.

[35] Kolody D , Hoyle S . 2015 Evaluation of tag mixing assumptions in western pacific ocean skipjack tuna stock assessment models. Fish. Res. **163** , 127–140. (10.1016/j.fishres.2014.05.008)

[36] Langley A , Million J . 2012 Determining an appropriate tag mixing period for the Indian Ocean yellowfin tuna stock assessment. In Fourteenth Work. Party Trop. Tunas.

[37] Bravington MV , Grewe PM , Davies CR . 2016 Absolute abundance of southern bluefin tuna estimated by close-kin mark-recapture. Nat. Commun. **7** , 1–8. (10.1038/ncomms13162) PMC5114523 27841264

[38] Bravington MV , Skaug HJ , Anderson EC . 2016 Close-kin mark-recapture. Stat. Sci. **31** , 259–274. (10.1214/16-STS552)

[39] Bravington M , Grewe P , Davies C . 2013 Close-kin update. CCSBT-ESC/1309/BGD 03. CSIRO. See https://www.ccsbt.org/system/files/resource/ja/521ebb122ae21/ESC18_BGD03_Australia_Close_kin.pdf. 10.1038/ncomms13162 PMC5114523 27841264

[40] Williams A , Hillary R , Preece A . 2021 Work plan for an Indian Ocean yellowfin tuna close-kin mark- recapture design study yellowfin tuna CKMR design study. csiro:EP2022-1478. CSIRO. (10.25919/r7e5-8c34)

[41] Williams A , Tremblay-boyer L , Hillary R . 2023 A close-kin mark-recapture pilot study for Indian Ocean yellowfin tuna. csiro:EP2023-5364. CSIRO. See http://hdl.handle.net/102.100.100/636556?index=1.

[42] Kolody D , Bravington M . 2019 Is close-kin mark recapture feasible for IOTC yellowfin tuna stock assessment. IOTC–2019–WPM10–25-rev. IOTC. See https://www.iotc.org/sites/default/files/documents/2019/10/IOTC-2019-WPM10-25_Rev1.pdf.

[43] McKinney R , Gibbon J , Wozniak E , Galland GG . 2020 Netting billions 2020: a global valuation of tuna. The Pew Charitable Trusts. See https://www.pewtrusts.org/-/media/assets/2020/10/nettingbillions2020.pdf.

[44] Moreno G , Dagorn L , Sancho G , Itano D . 2007 Fish behaviour from fishers’ knowledge: the case study of tropical tuna around drifting fish aggregating devices (DFADs). Can. J. Fish. Aquat. Sci. **64** , 1517–1528. (10.1139/f07-113)

[45] Guillotreau P , Salladarré F , Dewals P , Dagorn L . 2011 Fishing tuna around fish aggregating devices (FADs) vs free swimming schools: skipper decision and other determining factors. Fish. Res. **109** , 234–242. (10.1016/j.fishres.2011.02.007)

[46] Guillotreau P , Salladarré F , Capello M , Dupaix A , Floc’h L , Tidd A , Tolotti M , Dagorn L . 2024 Is FAD fishing an economic trap? Effects of seasonal closures and other management measures on a purse‐seine tuna fleet. Fish Fish. **25** , 151–167. (10.1111/faf.12799)

[47] IOTC-WPPT25 . 2023 report of the 25th session of the IOTC working party on tropical tunas. IOTC–2023–WPTT25–R[E. IOTC. See https://iotc.org/sites/default/files/documents/2023/12/IOTC-2023-WPTT25-RE.pdf.

[48] Matsumoto T , Yokoi H , Satoh K , Kitakado T . 2018 Diagnoses for stock synthesis model on yellowfin tuna in the Indian Ocean. IOTC–2018–WPTT20–42_Rev1. IOTC. See https://iotc.org/sites/default/files/documents/2018/10/IOTC-2018-WPTT20-42_Rev1.pdf.

[49] IOTC-SC26 . 2023 Report of the 26 th session of the IOTC scientific committee. IOTC-2023-SC26-R_Rev1. IOTC. See https://iotc.org/sites/default/files/documents/2024/06/IOTC-2023-SC26-R_Rev1E.pdf.

[50] Baidai Y , Dagorn L , Amande MJ , Gaertner D , Capello M . 2020 Machine learning for characterizing tropical tuna aggregations under drifting fish aggregating devices (DFADs) from commercial echosounder buoys data. Fish. Res. **229** , 105613. (10.1016/j.fishres.2020.105613)

[51] Capello M , Deneubourg JL , Robert M , Holland KN , Schaefer KM , Dagorn L . 2016 Population assessment of tropical tuna based on their associative behavior around floating objects. Sci. Rep. **6** , 36415. (10.1038/srep36415) 27808175 PMC5093414

[52] Ohta I , Kakuma S . 2005 Periodic behavior and residence time of yellowfin and bigeye tuna associated with fish aggregating devices around Okinawa islands, as identified with automated listening stations. Mar. Biol. **146** , 581–594. (10.1007/s00227-004-1456-x)

[53] Robert M , Dagorn L , Deneubourg JL , Itano D , Holland K . 2012 Size-dependent behavior of tuna in an array of fish aggregating devices (FADs). Mar. Biol. **159** , 907–914. (10.1007/s00227-011-1868-3)

[54] Govinden R , Capello M , Forget F , Filmalter JD , Dagorn L . 2021 behavior of skipjack (Katsuwonus pelamis), yellowfin (Thunnus albacares), and bigeye (T. obsesus) tunas associated with drifting fish aggregating devices (dFADs) in the indian ocean, assessed through acoustic telemetry. Fish. Oceanogr. **30** , 542–555. (10.1111/fog.12536)

[202304041] IOTC Secretariat . 2023 Instrumented buoy data (Jan 2020—December 2022). See https://iotc.org/sites/default/files/documents/2023/06/IOTC-2023-WGFAD04-DATA04_Rev1-BU.xlsx.

[56] Bach P , Cauquil P , Depetris M , Duparc A , Floch L , Lebranchu J , Sabarros P . 2018 Procédures d’échantillonnage des thonidés tropicaux débarqués par les senneurs dans les océans atlantique et indien. Sète: IRD. See https://horizon.documentation.ird.fr/exl-doc/pleins_textes/divers19-05/010075957.pdf.

[57] Duparc A , Aragno V , Depetris M , Floch L , Cauquil P , Lebranchu J , Gaertner D , Marsac F , Bach P . 2020 Assessment of the species composition of major tropical tunas in purse seine catches: a new modelling approach for the tropical tuna treatment processing (case of the French fleet in Atlantic Ocean). Collect. Vol. Sci. Pap. ICCAT **76(6)** , 951–982. https://www.iccat.int/Documents/CVSP/CV076_2019/n_6/CV076060951.pdf

[58] Depetris M , Lebranchu J . 2020 OB7-IRD/T3: beta version of T3 software (version 0.9.0). Zenodo. (10.5281/zenodo.3878125)

[59] Baidai Y , Dagorn L , Amandè MJ , Gaertner D , Capello M . 2020 Tuna aggregation dynamics at drifting fish aggregating devices: a view through the eyes of commercial echosounder buoys. ICES J. Mar. Sci. **77** , 2960–2970. (10.1093/icesjms/fsaa178)

[60] Pérez G *et al* . 2020 Effects of habitat modifications on the movement behavior of animals: the case study of fish aggregating devices (FADs) and tropical tunas. Mov. Ecol. **8** , 1–11. (10.1186/s40462-020-00230-w) 33292617 PMC7654007

[61] Rodriguez-Tress P , Capello M , Forget F , Soria M , Beeharry S , Dussooa N , Dagorn L . 2017 Associative behavior of yellowfin thunnus albacares, skipjack katsuwonus pelamis, and bigeye tuna T. obesus at anchored fish aggregating devices (FADs) off the coast of mauritius. Mar. Ecol. Prog. Ser. **570** , 213–222. (10.3354/meps12101)

[62] Robert M , Dagorn L , Lopez J , Moreno G , Deneubourg JL . 2013 Does social behavior influence the dynamics of aggregations formed by tropical tunas around floating objects? an experimental approach. J. Exp. Mar. Biol. Ecol. **440** , 238–243. (10.1016/j.jembe.2013.01.005)

[63] Dagorn L , Pincock D , Girard C , Holland K , Taquet M , Sancho G , Itano D , Aumeeruddy R . 2007 Satellite-linked acoustic receivers to observe behavior of fish in remote areas. Aquat. Living Resour. **20** , 307–312. (10.1051/alr:2008001)

[64] Matsumoto T , Satoh K , Toyonaga M . 2014 Behavior of skipjack tuna (Katsuwonus pelamis) associated with a drifting FAD monitored with ultrasonic transmitters in the equatorial central pacific ocean. Fish. Res. **157** , 78–85. (10.1016/j.fishres.2014.03.023)

[65] Matsumoto T , Satoh K , Semba Y , Toyonaga M . 2016 Comparison of the behavior of skipjack (Katsuwonus pelamis), yellowfin (Thunnus albacares) and bigeye (T. obesus) tuna associated with drifting fads in the equatorial central pacific ocean. Fish. Oceanogr. **25** , 565–581. (10.1111/fog.12173)

[66] Tolotti MT , Forget F , Capello M , Filmalter JD , Hutchinson M , Itano D , Holland K , Dagorn L . 2020 Association dynamics of tuna and purse seine bycatch species with drifting fish aggregating devices (fads) in the tropical eastern atlantic ocean. Fish. Res. **226** , 105521. (10.1016/j.fishres.2020.105521)

[67] Robert M , Dagorn L , Filmalter JD , Deneubourg JL , Itano D , Holland K . 2013 Intra-individual behavioral variability displayed by tuna at fish aggregating devices (fads). Mar. Ecol. Prog. Ser. **484** , 239–247. (10.3354/meps10303)

[68] Dagorn L , Bez N , Fauvel T , Walker E . 2013 how much do fish aggregating devices (fads) modify the floating object environment in the ocean?. Fish. Oceanogr. **22** , 147–153. (10.1111/fog.12014)

[69] Baidai Y *et al* . 2022 A standard processing framework for the location data of satellite-linked buoys on drifting fish aggregating devices. Aquat. Living Resour. **35** , 13. (10.1051/alr/2022013)

[70] Davies TK , Mees CC , Milner-Gulland EJ . 2014 Modelling the spatial behaviour of a tropical tuna purse seine fleet. PLoS One **9** , 1–18. (10.1371/journal.pone.0114037) PMC4252080 25462165

[71] Fromentin JM , Fonteneau A . 2001 Fishing effects and life history traits: a case study comparing tropical versus temperate tunas. Fish. Res. **53** , 133–150. (10.1016/S0165-7836(00)00299-X)

[72] Fréon P , Dagorn L . 2000 Review of fish associative behaviour toward a generalisation of the meeting point hypothesis. Rev. Fish Biol. Fish. **10** , 183–207. (10.1023/A:1016666108540)

[73] Rigby C , Sherman CS , Chin A , Simpfendorfer C . 2017 Carcharhinus falciformis. IUCN Red List Threat. Species 2017 e.T39370A1. (10.2305/IUCN.UK.2017-3.RLTS.T39370A117721799.en)

[74] Rigby C *et al* . 2019 Carcharhinus longimanus. IUCN Red List Threat. Species 2019 e.T39374A2. (10.2305/IUCN.UK.2019-3.RLTS.T39374A2911619.en)

[75] Forget FG , Capello M , Filmalter JD , Govinden R , Soria M , Cowley PD , Dagorn L . 2015 Behaviour and vulnerability of target and non-target species at drifting fish aggregating devices (FADs) in the tropical tuna purse seine fishery determined by acoustic telemetry. Can. J. Fish. Aquat. Sci. **72** , 1398–1405. (10.1139/cjfas-2014-0458)

[76] Evans PGH . 1982 Associations between seabirds and cetaceans: a review. Mamm. Rev. **12** , 187–206. (10.1111/j.1365-2907.1982.tb00015.x)

[77] Proctor H , Owens I . 2000 Mites and birds: diversity, parasitism and coevolution. Trends Ecol. Evol. **15** , 358–364. (10.1016/s0169-5347(00)01924-8) 10931667

[78] Wells RJD , Rooker JR . 2004 Spatial and temporal patterns of habitat use by fishes associated with sargassum mats in the northwestern gulf of mexico. Bull. Mar. Sci. **74** , 81–99.

[79] Griffin DC , Harrod C , Houghton JDR , Capellini I . 2019 Unravelling the macro-evolutionary ecology of fish–jellyfish associations: life in the ‘gingerbread house’. Proc. R. Soc. B. **286** , 20182325. (10.1098/rspb.2018.2325) PMC6452070 30890095

[80] Hoffman W , Fritts TH , Reynolds RP . 1981 Whale sharks associated with fish schools off south texas. N. E. Gulf Sci. **5** , 8–11. (10.18785/negs.0501.08)

[81] Baidai Y , Dupaix A , Gaertner D , Dagorn L , Deneubourg JL , Duparc A . 2024 Data from: Direct assessment of tropical tuna abundance from their associative behavior around floating objects. Figshare. (10.6084/m9.figshare.c.7403389)39163978

